# Profile of Rat Adrenal microRNAs Induced by Gonadectomy and Testosterone or Estradiol Replacement

**DOI:** 10.3390/ijms26104543

**Published:** 2025-05-09

**Authors:** Karol Jopek, Marianna Tyczewska, Małgorzata Blatkiewicz, Anna Olechnowicz, Marta Szyszka, Ewelina Stelcer, Sylwia Ciesiółka, Maria Jopek, Ludwik K. Malendowicz, Marcin Ruciński

**Affiliations:** 1 Department of Histology and Embryology, Poznan University of Medical Sciences, 60-781 Poznan, Poland; karoljopek@ump.edu.pl (K.J.); maritycz@ump.edu.pl (M.T.); mblatkiewicz@ump.edu.pl (M.B.); aolechnowicz@ump.edu.pl (A.O.); mszyszka@ump.edu.pl (M.S.); sciesiolka@ump.edu.pl (S.C.); m.kociecka@gmail.com (M.J.); marcinruc@ump.edu.pl (M.R.); 2 Department of Anatomy and Histology, University of Zielona Gora, Licealna Street 9, 65-417 Zielona Gora, Poland; 3 Department of Biochemistry and Biotechnology, Poznan University of Life Sciences, 60-637 Poznan, Poland; ewelina.stelcer@up.poznan.pl

**Keywords:** adrenal cortex, miRNA, sex differences, gonadectomy, gonadal hormone replacement

## Abstract

Sex-related differences in the structure and function of the adrenal cortex in mature rats are well recognized, largely driven by the action of sex hormones on the hypothalamic–pituitary–adrenal axis (HPA). By replacing testosterone or estradiol in gonadectomized rats, we aimed to elucidate the regulation of micro RNA (miRNA) profiles by sex hormones and their role in physiological adrenal function, providing new insights into gene expression modulation in the adrenal gland. This paper focuses on the description of miRNA profiles using the microarray technique. In our study, we observed significant sex differences in miRNA and mRNA expression levels. These differences are as follows: miRNA expression profiles Male C vs. Female C-0 down, 25 up-regulated, while mRNA profiles were 43 down and 27 up-regulated. Moreover, we observed the most significant differences in miRNA profiles between orchiectomized male rats supplemented with testosterone (ORX + T) and ovariectomized female rats treated with estradiol (OVX + E). Furthermore, we described changes in target gene expression and biological processes regulated by miRNAs. The processes most differentially expressed between the ORX + T and OVX + E groups are those related to the metabolism and synthesis of sterol compounds, the positive and negative regulation of metabolic processes in cells, e.g., cholesterol metabolism, response to various external factors, e.g., hormones, regulation of processes related to cell motility. We also identified several miRNAs, such as miR-370, miR-377, and miR-503, that exhibited interesting changes in their expression after testosterone or estradiol replacement. These results contribute to a deeper understanding of adrenal physiology.

## 1. Introduction

Sex-related differences in the structure and function of the adrenal cortex of mature rats are well recognized. These differences are evoked by the action of sex hormones on the HPA. Many studies showed that estrogens stimulate, and androgens inhibit HPA functions [1,2,3,4,5]. In our previous studies, we analyzed the effect of gonadectomy and testosterone and estradiol replacement on the expression of differentially regulated genes in the rat adrenal gland by microarray technique [6,7]. We demonstrated that in orchiectomized rats (ORX), testosterone replacement stimulates expression of numerous genes, mainly those associated with lipids and cholesterol metabolism. However, in ovariectomized animals (OVX), estradiol replacement inhibits the expression of genes, mainly those involved in intracellular signaling pathways. These findings have brought some new insights into the molecular basis of sex hormones’ action on the adrenal glands. The experimental model we used provides data suggesting a physiological role for androgens and estrogens in regulating rat adrenal cortex function.

As is currently known, the regulation of gene expression occurs through a variety of factors, among which miRNAs play an essential role. miRNAs are evolutionarily conserved, endogenous, non-coding RNAs approximately 20–23 nucleotides in length. These molecules usually have an uridine at the 5′-end, and they are partially complementary to mRNA molecules (target mRNAs) [8]. Their main function is to downregulate gene expression. The mechanism of action is based on the binding of mature miRNA to the target mRNA, usually within the 3′-untranslated region (3′-UTR). In situations when the complementarity of the mRNA to the mature miRNA is complete or almost complete, transcript degradation is observed. On the other hand, incomplete complementarity causes inhibition of translation [9,10,11,12]. miRNAs are classified in groups called miRNA families. A family of miRNAs contains molecules that possess a common origin, conserved sequence, and relationships with target mRNAs. [13,14]. The function of genes in the miRNA family is considered to be common, and it has been observed that miRNAs in the same miRNA family are co-localized and well organized around genes involved in many diseases, development, and cancer [15,16,17,18,19,20,21,22]. Moreover, miRNAs regulate processes of the immune responses, differentiation, tumorigenesis, cell death, and neurodegeneration [23,24,25,26,27,28,29]. Additionally, miRNAs can act as tumor suppressors or oncogenes [30,31,32].

MicroRNAs have also been detected in adrenal glands, and some studies have identified specific miRNAs that show altered levels in adrenal tumors, affecting various signaling pathways involved in key cellular processes such as cell proliferation and apoptosis [33,34,35,36,37,38,39]. For example, miR-483-5p and miR-34a are candidate serum biomarkers for differentiating between benign and malignant adrenocortical tumors with accuracies of 74% and 81%, respectively [40]. Additionally, elevated serum levels of miR-483-5p or diminished levels of miR-195 in adrenocortical carcinoma patients have been linked to both reduced recurrence-free survival and decreased overall survival [41].

In contrast to human adrenal glands, especially with regard to their pathology, the expression of miRNAs in rat adrenal glands is poorly understood. We found only one report in the literature on the expression of miRNAs in adult intact male rats treated with ACTH, dexamethasone, and estradiol [42]. The authors have demonstrated that the experimental conditions applied (stimulation or inhibition of adrenal cortex function) significantly modify the expression profile of various miRNAs. In light of these studies, we decided to investigate the effects of androgens and estrogens on the expression profile of miRNAs in the adrenals of gonadectomized rats and replaced with testosterone or estradiol, respectively.

## 2. Results

To investigate the sex hormones’ modulatory effect on the rat adrenal miRNA profile, adult females and males were gonadectomized and replaced with estradiol or testosterone, respectively. The effectiveness of the treatments was confirmed by changes in seminal vesicles and uterine weight, as we published earlier [7]. The rats were divided into six experimental groups: sham-operated female and male (C), OVX, ORX, OVX + E, and ORX + T. A total of eighteen rats were utilized (three rats per group).

**Figure 1:**
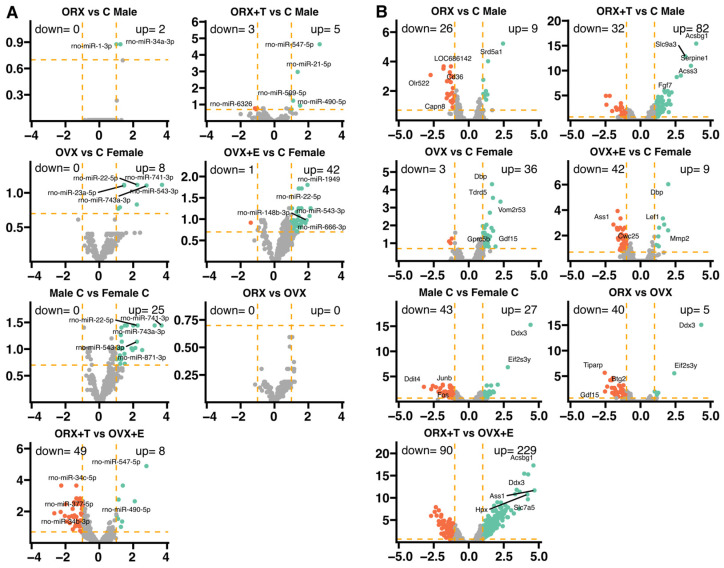
miRNA (**A**) and mRNA (**B**) transcriptome profiling of adrenals from adult male (Male C), female (Female C), orchiectomized male (ORX), ovariectomized female (OVX), orchiectomized with testosterone replaced male (ORX + T), and ovariectomized with estradiol replaced female (OVX + E) rats. Each dot on the volcano plot represents an average normalized expression of a single gene. Orange dashed lines are placed at the cut-off values (abs (fold) > 2 and *p* < 0.05 with 20% false discovery rate correction). Red dots are down-regulated genes, while green dots are up-regulated. Five of the most regulated genes are described by their gene symbols (if presented).

Adrenal glands taken from three rats per group were used for miRNA profiling by the Affymetrix microarray method. Transcriptome profiles were compared with sham-operated (control) adrenals (ORX vs. C Male, ORX + T vs. Male, OVX vs. C Female, OVX + E vs. C Female), and variations in miRNA expression were also compared among other groups (Male C vs. Female C, ORX vs. OVX, ORX + T vs. OVX + E). To identify miRNA target genes, we performed analogous comparisons of transcriptome mRNA expression for the same pairwise comparisons as for miRNAs, using previously published data [7]. For both miRNAs and mRNAs, we used the same cut-off criteria for differentially expressed genes/miRNAs (abs (fold change) > 2 and *p* < 0.05 with 20% FDR correction). General expression profiles were presented as volcano plots (Figure 1A). Taking into account the established cut-off criteria, we obtained two miRNA profiles in which there were no or a few significantly regulated miRNAs (ORX vs. C Male-0 down, 2 up-regulated; ORX vs. OVX-0 down, 0 up-regulated). Other comparisons were characterized by slight but stronger regulation of miRNA expression than previously described groups (ORX + T vs. C Male-3 down, 5 up-regulated; OVX vs. C Female-0 down, 8 up-regulated). The strongest miRNA regulation was obtained in the following comparisons: OVX + E vs. C Female-1 down, 42 up-regulated; Male C vs. Female C-0 down, 25 up-regulated; ORX + T vs. OVX + E-49 down, 8 up-regulated. It is worth noting that the overall miRNA expression profiles in the comparisons of OVX + E vs. C Female, C Male vs. C Female, ORX + T vs. OVX + E are inversely proportional to the respective mRNA profiles (OVX + E vs. C Female-miRNA: 1 down, 42 up regulated, mRNA: 42 down, 9 up regulated; C Male vs. C Female-miRNA: 0 down, 25 up regulated, mRNA: 43 down, 27 up regulated; ORX + T vs. OVX + E-miRNA: 49 down, 8 up regulated, mRNA: 90 down, 233 up regulated) (Figure 1B). The strongest effect on the miRNA and mRNA expression changes was observed in the ORX + T vs. OVX + E comparison.

**Figure 2:**
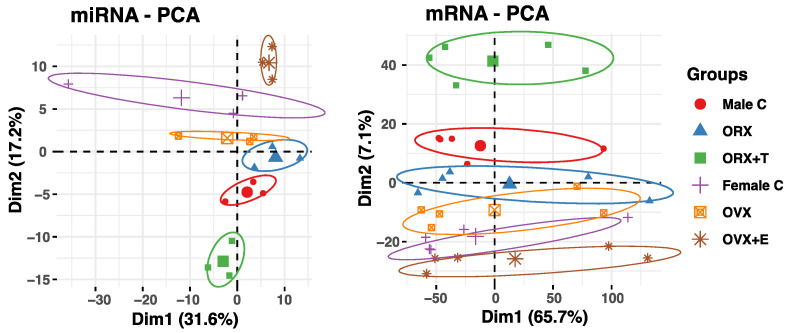
Principal component analysis (PCA) plots showing the first two principal components of the filtered miRNA (left panel) and mRNA (right panel) expression datasets. The datasets were reduced to two dimensions for visualization: Dimension 1 (Dim1) and Dimension 2 (Dim2). These dimensions correspond to Principal Component 1 and Principal Component 2, respectively.

The obtained results were then confirmed using Principal Component Analysis (PCA) for miRNA and mRNA expression data (Figure 2). For both datasets, PCA showed the strongest separation between the OVX + E and ORX + T, while the expression profiles in the orchidectomy and ovariectomy were the most similar and grouped together.

The obtained results were then confirmed using Principal Component Analysis (PCA) for miRNA and mRNA expression data (Figure 2). For both datasets, PCA showed the strongest separation between the OVX + E and ORX + T, while the expression profiles in the orchidectomy and ovariectomy were the most similar and grouped together.

**Figure 3:**
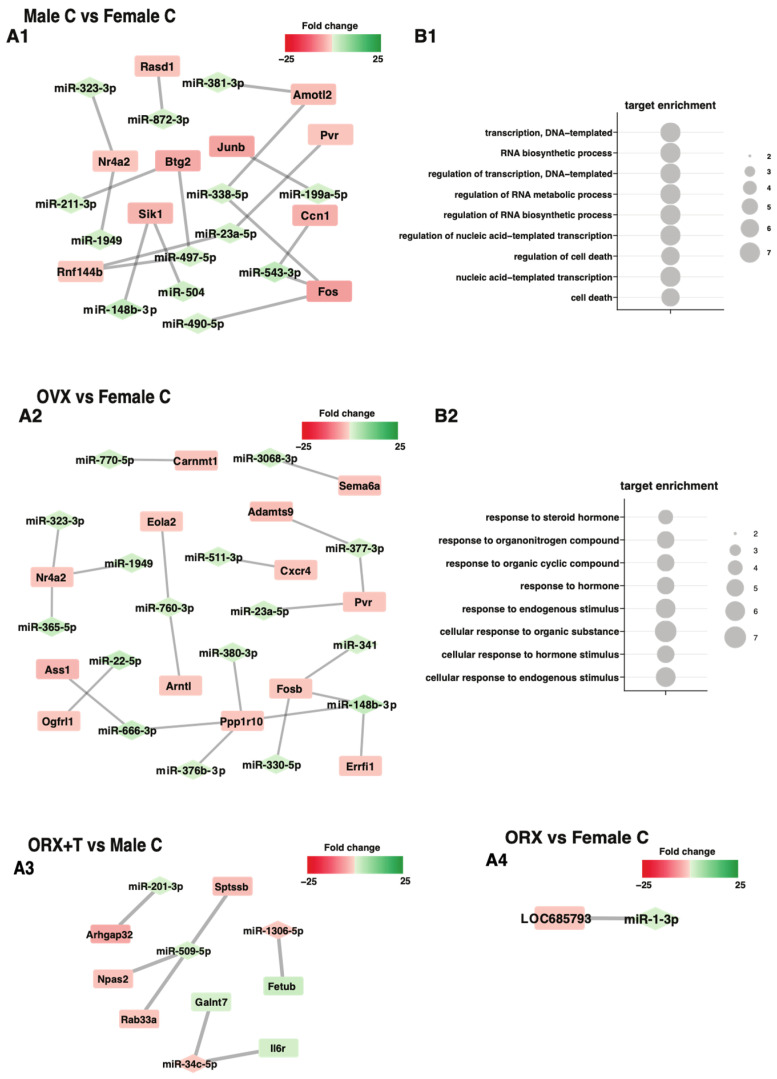
The graph shows the ‘miRNAtap’ package method in several groups of subjects: Male C vs. Female C, OVX vs. C Female, ORX + T vs. C Male, ORX vs. Female C. Only target genes above the cut-off criterion for differentially expressed genes were selected (abs (fold) > 2 *p* < 0.05 with 20% FDR correction). For the final identification of miRNA target genes, only those targets whose fold change was inversely correlated with the fold change in the corresponding miRNAs were left (**A1**,**A2**,**A3**,**A4**). The set of unique target genes thus selected was clustered using the DAVID bioinformatics tool to assign a specific GO term. Significant GO groups with adjusted *p*-values below 0.05 were visualized using a bubble plot (**B1**,**B2**). The changes in miRNA and mRNA expression have been color-coded according to the legend, where green indicates an increase in expression, and red indicates a decrease.

**Figure 4:**
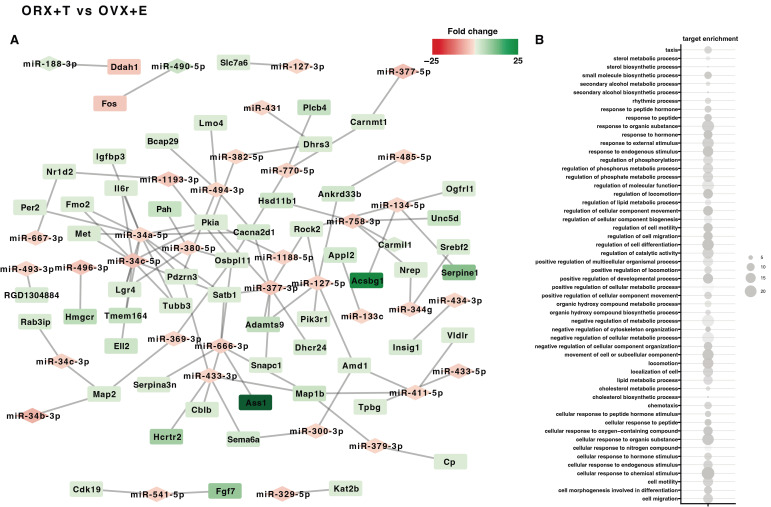
The graph shows the ‘miRNAtap’ package method in ORX + T vs. OVX + E group of subjects. Only target genes above the cut-off criterion for differentially expressed genes were selected (abs (fold) > 2 *p* < 0.05 with 20% FDR correction). For the final identification of miRNA target genes, only those targets whose fold change was inversely correlated with the fold change in the corresponding miRNAs were left (**A**). The set of unique target genes thus selected was clustered using the DAVID bioinformatics tool to assign a specific GO term. Significant GO groups with adjusted *p*-values below 0.05 were visualized using a bubble plot (**B**). The changes in miRNA and mRNA expression have been color-coded according to the legend, where green indicates an increase in expression, and red indicates a decrease.

Then, using the ‘miRNAtap’ package method, target prediction and miRNA–mRNA pairing were performed in groups based on previous results (Figure 1). As shown in Figure 3 and Figure 4, pairing was only successful in several study groups: Male C vs. Female C, OVX vs. C Female, ORX + T vs. C Male, ORX vs. Female C, and ORX + T vs. OVX + E. In the four groups, it was possible to find more than a dozen miRNA–mRNA pairs; only in the group ORX vs. Female C, one miRNA–mRNA pair was found (Figure 3(A1,A2) and Figure 4A-left side of the graph). In the case of three groups (Male C vs. Female C, OVX vs. C Female, and ORX + T vs. OVX + E), the pairs studied managed to assign specific ontological processes (Figure 3(B1,B2) and Figure 4B-right side of the graph). The ontological processes in Male C vs. Female C are mainly the processes of regulation of DNA and nucleic acid transcription, RNA synthesis and metabolism, and regulation of cell death (Figure 3). While the ontological processes in the OVX vs. Female C group are processes related to the response to various external factors, e.g., steroid hormones, organic compounds, or intrinsic stimulants (Figure 3). Both groups show increased expression of miRNA molecules and decreased expression of mRNA.

The highest number of miRNA–mRNA pairs and the highest number of assigned ontology groups were obtained in the ORX + T vs. OVX + E group (Figure 4). In the graph, it can be seen that miRNA molecules show, for the most part, down-regulated expression, while mRNA molecules show up-regulated expression, the opposite of the pairs in the previous groups. Among the ontological processes, the predominant ones are those related to metabolism and synthesis of sterol compounds, positive and negative regulation of metabolic processes in cells, e.g., cholesterol metabolism, response to various external factors, e.g., hormones, and regulation of processes related.

Next, we analyzed the expression patterns in each group using the K-means clusterization algorithm. For the clusterization, we extract only miRNA expression data whose expression was significantly regulated in at least one of the compared pairs. First, we established the optimal number of clusters using the sum of squared error (SSE) approach with an increasing number of clusters. Using this approach, we determined the optimal number of clusters as two. For each cluster, we determined the centroid values and core miRNA sets. According to our assumptions, miRNAs belonging to a particular cluster are required to have a high level of correlation to centroid values for a given cluster (correlation > 0.8). The first cluster included miRNAs with the highest expression in the ORX + T group. The expression of these genes was also slightly higher in males in Male C, ORX groups relative to all female groups. The second cluster was characterized by the lowest miRNA expression in the ORX + T with the highest expression in the ORX + E group.

**Figure 5:**
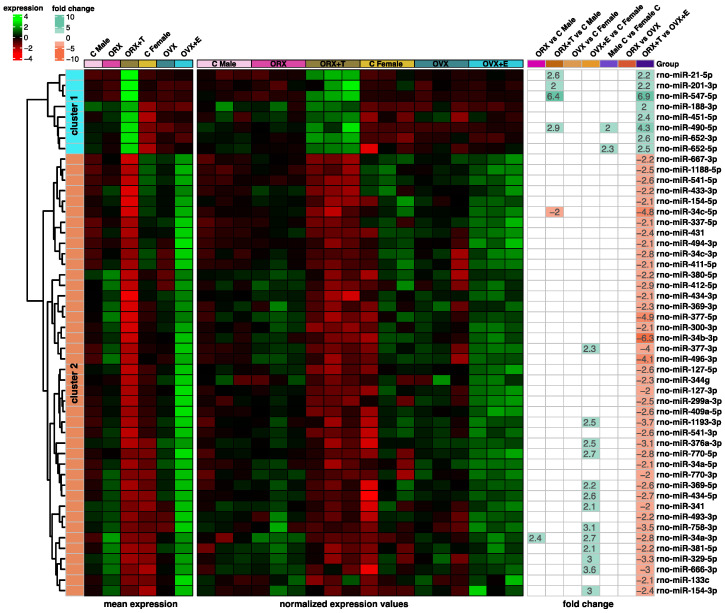
Heatmap of two miRNA clusters obtained from the K-means clustering algorithm. The heatmap comprises three panels described at the bottom of the graph. The “mean expression” and “normalized expression values” panels contain the mean or raw expression data scaled by rows. The “fold change” panel contains the fold change values according to the presented scale.

The obtained miRNA expression clusters were visualized as a heatmap (Figure 5) where hierarchical clustering was performed within the miRNAs of the first cluster (N = 8, turquoise color) and the second cluster (N = 42, orange color). The figure shows both mean expression values, normalized expression values, and fold changes between the compared groups. When considering the mean expression values and the normalized expression values panels, in the first cluster, a clear increase in the expression of all analyzed miRNAs is observed in the ORX + T group. In the other groups, expression levels show significantly lower values, especially in the Female C, OVX, and OVX + E groups. In cluster two, the expression profile of miRNA molecules is rather opposite. A large decrease in expression can be observed in the ORX + T group, and an increase, especially in the OVX + E group. This is evident both by looking at the mean expression values and the normalized values panels. The third panel of the graph, fold change, highlights the expression levels of miRNA molecules when the different study groups were compared with each other. It can be observed that the greatest changes in expression are for molecules in the ORX + T vs. OVX + E group comparison. In cluster one, there was a significant increase in expression, while in cluster two, there was a significant decrease, according to the scale presented. For the other comparisons between groups, significant differences were still only seen in the ORX + T vs. Male C and OVX + E vs. Female C and Male C vs. Female C groups.

**Figure 6:**
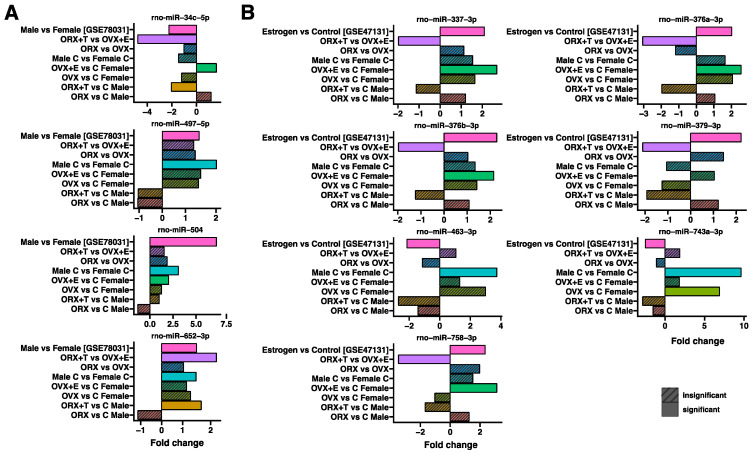
Analysis of common miRNAs regulated in our study and in the available rat adrenal transcriptomic databases GSE78031 (**A**) and GSE47131 (**B**). Expression profiles are shown for all comparisons in Figure 1. Statistical significance is shown as a smooth (statistically significant) or hatched (non-significant) rectangle, according to the legend. Each chart takes into account the corresponding fold-change in expression.

Figure 6 shows an analysis of the common miRNAs regulated in our study and in the available rat adrenal transcriptomic databases (GSE78031, GSE47131); therefore, the presented miRNAs are not necessarily those that accumulate the largest number of target genes in our study. In part A, the expression of specific miRNA molecules was compared in the study groups in relation to the Male vs. Female group (GSE78031), and in part B-to the Estrogen vs. Control group (GSE47131). It is clearly visible that the expression profile of the analyzed miRNA molecules is, in most cases, consistent with the data available in the rat adrenal transcriptomic databases, although not always statistically significant. The expression profile of rno-miR-652-3p is the most similar among the analyzed miRNA sets. The least similar expression profile is shown by rno-miR-379-3p, rno-miR-743a-3p, rno-miR-463-3p, and rno-miR-758-3p.

**Figure 7:**
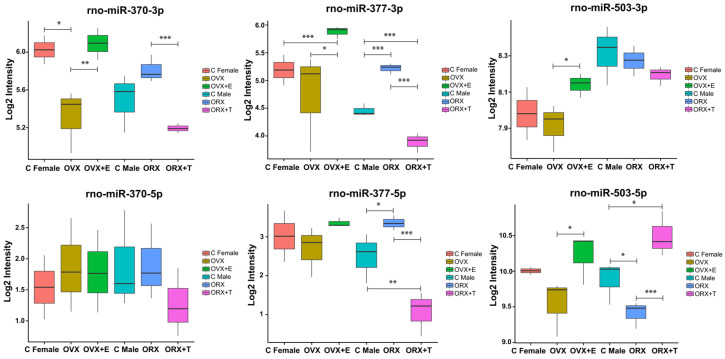
Bar plots showing the results of microarray analysis of selected miRNAs in adrenals from adult male (Male C), female (Female C), orchidectomized male (ORX), ovariectomized female (OVX), orchidectomized with testosterone replaced male (ORX + T), and ovariectomized with estradiol replaced female (OVX + E) rats. The number of biological replicates was three per group. The significance levels are: *** <0.01, ** <0.05 and * <0.1.

As mentioned in the Introduction, in the only report on the expression of miRNAs in adult male rats treated with estradiol, Hu et al. demonstrated that the levels of miR-212, miR-183, miR-182, miR-132, miR-370, miR-377, and miR-96 were upregulated, whereas miR-125b, miR-200b, miR-122, miR-466b, miR-138, miR-214, miR-503 and miR-27a were downregulated in response to 17α-E2 treatment. Analyzing the data published by Hu et al. from the results we obtained, we selected the following miRNAs for further detailed analyses: miR-370, miR-377, and miR-503. The results of these analyses are shown in Figure 7.

**Figure 8:**
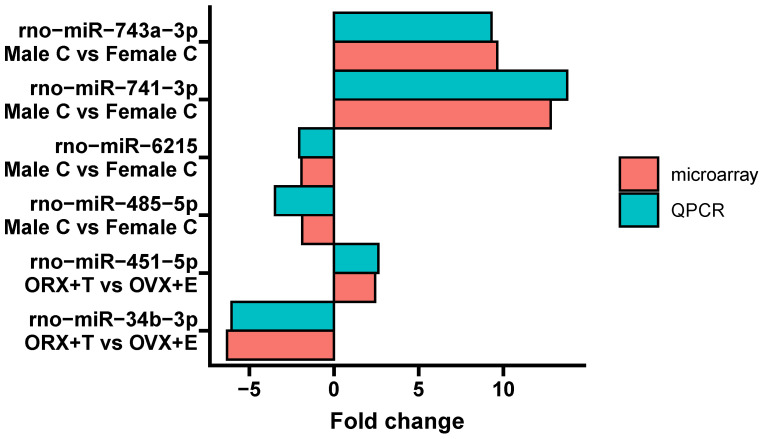
A bar plot showing the results of qPCR analysis of miRNAs, compiled with results from microarray analysis. The number of biological replicates was three per group. The expression of the studied miRNAs was normalized to RNU1A1.

The last graph shows a comparison of the expression of selected miRNA molecules (rno-miR-743a-3p, rno-miR-741-3p, rno-miR-6215, rno-miR-458-5p, rno-miR-451-5p, and rno-miR-34b-3p) obtained by matrix analysis and QPCR as a validation method (Figure 8). That analysis clearly indicates that the expression profile coincides exactly, both in terms of an increase or decrease in expression, and the statistical significance of individual molecules.

## 3. Discussion

miRNAs play a crucial role in the regulation of adrenal steroidogenesis and the pathogenesis of adrenal tumors [38]. A growing body of literature indicates that many miRNA molecules might affect adrenal cells in different ways. For instance, in the human adrenal cortex, miR-24 was found to modulate the expression of 11-beta hydroxylase (Cyp11b1) and aldosterone synthase (Cyp11b2), leading to changes in aldosterone and cortisol secretion rates [43]. Similarly, miR-21 was shown to stimulate aldosterone secretion and promote proliferation in angiotensin-II-responsive steroid-producing adrenocortical cell line (H295R cells) [44]. On the other hand, miR-132 was found to inhibit steroidogenesis in Y1 adrenocortical cells. Moreover, upon hormonal stimulation, miR-132 induces the expression of 3β-hydroxysteroid dehydrogenase (3β-Hsd) and 20-α-hydroxysteroid dehydrogenase (20α-Hsd) by inhibiting the transcriptional factor MeCP2 [45].

Gender-related differences in adrenal cortex structure and function in mature rats are highly significant and now well known. These differences are caused by the action of sex hormones on the hypothalamic–pituitary–adrenal (HPA) axis. Numerous scientific reports indicate that estrogens could stimulate, while androgens might inhibit HPA function [3,7,46,47,48]. To date, no research studies have investigated the impact of gonadectomy and sex hormone replacement on the regulation of miRNA expression in the adrenal glands of rats. However, we previously explored the impact of gonadectomy and the associated testosterone and estradiol replacement on the gene expression profile of differentially regulated genes in rat adrenal glands [7]. The findings revealed novel insights into the molecular mechanisms underlying the effects of sex hormones on the adrenal glands. Notably, testosterone replacement resulted in the potent stimulation of the expression of numerous adrenal genes, particularly those involved in lipid and cholesterol metabolism. In contrast, estradiol replacement induced a marked down-regulation of genes associated with the regulation of TNFA signaling via NFKB pathways [7]. The study by Jopek et al. [7] did not analyze the experimental groups ORX vs. OVX, Male C vs. Female C, or ORX + T vs. OVX + E, which were examined in the present study. Herein, we found that among the ORX vs. OVX group, the expression of 40 mRNAs was up-regulated, and 5 were down-regulated. In the ORX + T vs. OVX + E group, there were changes in the expression of over 300 mRNAs (90 down-regulated and 229 up-regulated). When looking at the ontological processes that were regulated to the greatest extent, we found processes related to the metabolism and synthesis of sterol compounds, as well as positive and negative regulation of cholesterol metabolism, similar to the previously indicated GO processes.

Building upon these findings, we extended our investigation to assess the effect of testosterone and estradiol on the mRNA and miRNA transcriptome profile of rat adrenal glands. Hence, to gain a deeper understanding of this problem, the same experimental model was used (removal of the gonads and replacement with appropriate hormones) as in the previous study [7]. As mentioned earlier, microarray analysis was conducted using three adrenal glands from each study group. The comparison between the experimental groups resulted in the identification of two distinct miRNA expression profiles. The first profile consisted of a small number of up-regulated miRNAs. For example, the comparison between ORX vs. C Male (0 down and only 2 up-regulated) group. The second profile, on the other hand, showed miRNAs with a small but significantly stronger regulation of expression. For instance, the ORX + T vs. C Male group had three down-regulated and five up-regulated miRNAs. Moreover, the most significant miRNA regulation was observed in the comparisons between the adrenal glands of the male and female, and between the orchidectomy group treated with testosterone versus the ovariectomy group treated with estradiol. The findings suggest, therefore, that the dissimilarities between the adrenal glands of the rats could mainly be attributed to variances in gender. Furthermore, it is noteworthy to underline that the differences observed between the study groups are inversely related to the corresponding mRNA profiles of these groups. The above results were confirmed using the PCA method, where it is clear that the largest discrepancies were observed for the ORX + T vs. OVX + E groups.

While no analogous literature data exists for the adrenal glands, comparable information concerning differentially expressed miRNAs is accessible in the literature for other organs and tissues. Link et al. conducted an intriguing study that examined the impact of gonadal hormones and sex chromosome composition on the miRNA expression profile in adipose tissue [49]. The findings revealed that miRNA expression profiles were modified in response to gonadectomy and a high-fat diet. In their study, they paid special attention to two miRNAs: miR-192-5p and miR-205-5p. Notably, the study reported that the levels of miR-192-5p were greater in males than in females of mice with intact gonads. However, this sex disparity disappeared after gonadectomy. Additionally, miR-205-5p showed a sex chromosome bias (XX > XY) in intact mice with gonads, and a sex bias (male > female) in mice after gonadectomy and fed with a chow diet [49]. As they noted, diverse miRNA expression profiles may contribute to sex differences in adipose tissue gene expression, adipose tissue development, and diet-induced obesity.

Wang et al. presented a noteworthy study in which they deleted Dicer in gonadotrophs, leading to suppression of gonadotropin synthesis and secretion, and resulting in fertility defects in mice [50]. Specifically, miRNAs which bind to the mRNAs encoding three pituitary glycoprotein hormone subunits (Fshb, Lhb, and Cga)-such as mir-150-5p, mir331-3p, mir-361-3p, mir-515-5p, mir-516A-3p, mir-532-3p, mir-708-5p, mir-665-5p, mir-1273H-3p, mir-3127-5p, mir-4785, mir-362-5p, and mir-3173-3p-were significantly suppressed in gonadotrophs of Dicer conditional knockout mice. These findings confirm that targeted deletion of Dicer in gonadotrophs leads to the loss of miRNAs that regulate gonadotropin β subunit-encoding mRNAs and the secretion of heterodimeric hormone [50].

In our study, we found the highest number of miRNA–mRNA pairs and ontology groups in the ORX + T vs. OVX + E group. Among the ontological processes, those related to the metabolism and synthesis of sterol compounds, positive and negative regulation of cellular metabolic processes, response to various external factors, and regulation of processes related to cell motility were most prevalent. We conducted K-means clustering to investigate this information further and identified two distinct groups of miRNAs. The first group showed significant up-regulation of expression, while the second group showed significant down-regulation. Subsequent analyses revealed that the second cluster had a significantly higher number of target genes (regulated by 34 miRNA molecules) than the first. Notably, miR-496-3p, miR-344g, miR-770-5p, and miR-299a-3p were among the molecules with the highest number of target genes.

As the miRNA molecules in cluster two of our study regulated a large number of target genes, we conducted additional enrichment analysis using the DAVID GO BP FAT database to gain further insight into the ontological processes involved. Our analysis revealed that these miRNAs were primarily involved in processes related to the response to organic substances and chemical stimulants, as well as the regulation of phosphorus metabolism, phosphate processes, and phosphorylation. Additionally, these miRNAs were found to regulate processes related to cell movement, cell localization, and cell differentiation. On the other hand, the most significant changes in expression were observed in ontological processes related to cellular response to organic substances, such as hormones, as well as processes regulating catalytic activity, metabolic processes, protein modification, and cellular localization. It is worth noting that the down-regulation of miRNA expression observed was associated with up-regulation of mRNA molecules, which supports previous literature on this topic.

Noticeable are the results obtained by Noutsios et al., who investigated miRNA differences in male and female transgenic mice expressing surfactant protein 2 (SPA2) after undergoing gonadectomy [51]. Considering that innate defense molecules in humans, specifically surfactant protein A1 and A2 (SP-A1 and SP-A2), have varying effects on the function and proteome of alveolar macrophages (AM), they conducted a study to examine the potential impact of these molecules on gender-related differences in miRNA expression in mice. The study found that sex hormones played a significant role in regulating miRNA expression. Specifically, the expression of two miRNAs (5.4%) was significantly upregulated in the gonadectomy group compared to the non-gonadectomy group, while the expression of 34 miRNAs (94.6%) was significantly inhibited. Of the 37 miRNAs that were differentially expressed in males and females after gonadectomy, 17 miRNAs (45.9%) were specific to females after ovariectomy, and 7 miRNAs (18.9%) were specific to males after orchidectomy [51]. The researchers have confirmed their hypothesis that the studied protein plays an essential role in the gender-specific expression of miRNA molecules, and their research seems to support the significance of sex differences in miRNA expression.

Since there was a dearth of literature data on the expression profile changes in the miRNA molecules in rat adrenal glands, we conducted a comparative analysis of common and shared miRNAs, utilizing available rat adrenal transcriptomic databases, namely GSE78031 and GSE47131. Several common molecules were identified, including rno-miR-34c-5p, rno-miR-497-5p, rno-miR-504, rno-miR-652-3p, rno-miR-379-3p, rno-miR-743a-3p, rno-miR-463-3p, and rno-miR-758-3p. Among the analyzed miRNAs, the most similar expression profile was observed for rno-miR-652-3p and rno-miR-504, while the least similar expression profile was found for rno-miR-379-3p, rno-miR-743a-3p, rno-miR-463-3p, and rno-miR-758-3p. However, it should be noted that these miRNAs were not necessarily the ones that accumulated the highest number of target genes in our study. This is why the expression profiles of selected up-regulated miRNAs were confirmed by real-time qPCR. The results obtained showed a high degree of agreement, as observed for both miRNAs in the comparison of the control groups (males vs. females) and the experimental groups (ORX + T vs. OVX + E).

The comparative analysis between our results and those published by Hu et al. [42] revealed significant differences in the expression of miR-370, miR-377, and miR-503. Hu et al. reported that miR-370 and miR-377 were upregulated, while miR-503 was downregulated in response to 17α-E2 treatment in adult male rats. Additionally, miR-503 was also downregulated by ACTH. In our results, rno-miR-370-3p is upregulated by estradiol treatment in ovariectomized female rats but downregulated by testosterone treatment in orchidectomized male rats. It highlights a notable sex difference in the expression of miR-370. Existing literature suggests that miR-370 is dysregulated in various types of cancer, influencing critical biological processes such as cell proliferation, apoptosis, migration, invasion, and cell cycle progression. miR-370 may act as a tumor suppressor within the MEK/ERK pathway by regulating various target genes. miR-370 expression is downregulated in ovarian and cervical cancers, where it inhibits cell proliferation, invasion, migration, and cell cycle progression while promoting apoptosis [52]. Furthermore, miR-370 is recognized as a regulatory factor that reduces fat accumulation in the body. miR-370-3p expression was lower in the fat mass of mice on a high-fat diet compared to those on a normal control diet. Overexpression of miR-370-3p has been shown to significantly decrease the mRNA expression levels of adipogenic markers, thus reducing lipid accumulation. These findings suggest that miR-370-3p plays a critical role in adipogenesis and fatty acid metabolism [53]. It is worth mentioning that in our previous studies, we demonstrated that testosterone replacement in orchidectomized rats stimulates the expression of numerous genes, primarily those involved in lipid and cholesterol metabolism [7]. This observation may further support the role of miR-370-3p in lipid metabolism. Additionally, some studies indicate that low intrauterine levels of endogenous glucocorticoids may lead to persistent adrenal insufficiency in male offspring rats after birth. This effect is attributed to epigenetic programming of the GC-IGF1 axis, mediated by alterations in the GRα/miR-370-3p/Sirt3 pathway. In NCI-H295R cells treated with cortisol for 24 h, miR-370-3p expression was found to be decreased [54].

We observed that estradiol stimulates miR-377-3p expression, while testosterone inhibits it, revealing another interesting sex-based difference. Furthermore, testosterone suppresses miR-377-5p expression. Although limited data are available on the function of miR-377 in rat adrenals, its human homolog has been identified as a potential marker for distinguishing pediatric adrenocortical tumors from nonneoplastic adrenal tissues. Lower expression of miR-377 has been significantly associated with poor prognosis, tumor relapse, and/or death [55]. Further studies are needed to explore its role in rat adrenal function.

Finally, miR-503, the last miRNA in our comparative analysis, was shown to be downregulated by 17α-E2 or ACTH treatment in studies performed by Hu et al. [42]. In contrast, our results indicate that miR-503-5p is upregulated by testosterone treatment in orchidectomized rats, thus, miR-503-5p may play an important physiological regulatory function in the biological activity of the rat adrenal cortex. The function of miR-503 in adrenals remains unclear, but it has been suggested that its high expression correlates with shorter overall survival in patients with adrenocortical carcinoma [56].

The importance of miRNAs in adrenal cell function is supported by various studies that have identified molecules with prognostic significance for certain adrenal diseases, such as adult adrenocortical carcinoma (ACC) [34,57,58,59]. For example, miR-195 and miR-483-5p, which map to the second intron of the IGF2 gene and may be associated with the malignant phenotype of ACC, have been shown to have predictive value for patient outcomes [41,60,61,62]. It is worth mentioning that the levels of miR-483 and miR-483-5p correlate with the number of circulating tumor cells (CTCs) detected in the blood [40]. Additionally, miR-34a and miR-483-5p have the potential to become biomarkers for adrenocortical tumors since both are significantly elevated in the serum of ACC patients [63].

## 4. Materials and Methods

### 4.1. Animals and Experiments

Twelve-week-old male and female Wistar rats (body weight: 120–150 g) were received from the Laboratory Animals Breeding Center, Department of Toxicology, Poznan University of Medical Sciences, Poznan, Poland. All experimental procedures were approved by the Local Ethical Committee for Animal Research (Poznan, Poland), permit no: LKE-11/2015. Maximum possible care was taken to minimize the number of animals and their suffering. Rats were maintained under standard lighting conditions (light-dark cycle 14:10 h, light onset time: 06:00) at a constant temperature (23 °C) with free access to standard food and water. All experiments were conducted between 10 and 11 am. The rats were divided into six experimental groups (three rats per group): sham-operated female and male (C), OVX, ORX, OVX + E, and ORX + T. A total of eighteen rats were utilized. The data regarding the hormonal assessment of serum corticosterone levels, as well as the concentrations of total cholesterol, lipoproteins, and triglycerides in the same experimental groups, can be found in our previous report [7]. The present study focuses on transcriptomic alterations.

Rats were subjected to sham or gonadectomy surgery under anesthesia with ketamine (100 mg/kg, i.p.) and xylazine (10 mg/kg, i.p.). The detailed procedure was described previously [7]. ORX was performed via a scrotal access, while OVX was performed by two dorso-lateral incisions. Fourteen days after surgery, half of the ORX rats were replaced with testosterone (s.c. injection of Testoviron-Depot, Schering AG, Berlin, 5 mg/100 g body weight) while half of the OVX animals were given estradiol (s.c. injection of Estradiol-Depot, Jenapharm, 0.5 mg/100 g body weight). Doses of administered depo hormones were based on previous reports [46,47,48]. After 2 weeks (4 weeks after surgery), the rats were decapitated. Adrenal glands were collected and embedded in RNAlater (cat. No. R0901, Sigma-Aldrich, St. Louis, MO, USA), then stored at −70 °C for further analysis.

### 4.2. miRNA Isolation

The miRNAs were isolated using Qiagen’s miRNeasy Kit according to the manufacturer’s protocol (Cat. No. 217004, Qiagen, Hilden, Germany). The detailed procedure was described earlier [27,28,64]. The quality and concentration of isolated miRNA were quantified by measuring the absorbance at 260 nm and 280 nm. The degree of protein contamination was measured by estimating the absorbance ratio at 260/280 nm. The 260/280 nm ratio oscillated between 1.8 and 2.0 in all samples. From each group, three samples were selected for miRNA expression profiling by microarray (N/group = 3).

### 4.3. Microarray Preparation, Hybridization, and Scanning

miRNA expression profiling was carried out using miRNA expression microarrays based on the Affymetrix GeneAtlas platform with the GeneChip miRNA 4.1 Array Strip (ThermoFisher Scientific, Waltham, MA, USA). The microarrays were designed according to miRBase Release 20, containing complementary probes for 728 rat mature miRNA, 490 pre-miRNA. The isolated miRNA was modified before hybridization by the FlashTagTM Biotin HSR RNA Labeling Kit (ThermoFisher Scientific, Waltham, MA, USA). During this process, 150 ng of miRNA was subjected to poly (A) tailing and biotin labeling according to the manufacturer’s protocol. The biotin-labeled miRNA was subsequently hybridized to GeneChipTM miRNA 4.1 Array Strip (20 h, 48 °C). The microarrays were washed and stained according to the technical protocol using Affymetrix GeneAtlas Fluidics Station (Affymetrix, Santa Clara, CA, USA). Array strips were scanned by the GeneAtlas System Imaging Station (Thermo Fisher Scientific, Waltham, MA, USA). The quality of miRNA expression data was checked according to quality control criteria provided by the AffymetrixGeneAtlasTM Operating Software (version 2.0). Obtained CEL files obtained were imported into the downstream data analysis.

### 4.4. Microarray Data Analysis

All bioinformatics analyses were performed using BioConductor software (release 3.15, https://bioconductor.org) with relevant BioConductor packages, functioning as an extension of the R programming language (ver. 4.2.1; R Core Team 2022). Raw CEL files were normalized, background corrected, and summarized through the Robust Multiarray Averaging (RMA) algorithm implemented in the “affy” BioConductor package [65]. To assign microarray quality and diagnose batch effects, we used the “arrayQualityMetrics” package [66]. Biological annotation of rat miRNA was retrieved from the pd.mirna 4.1 package, where tabulated biological descriptors were merged with the normalized miRNA dataset to obtain the full miRNA data table. miRNAs with low variance in all samples were removed using the “genefilter” package [67]. Differential expression and statistical evaluation were calculated using the “limma” package, based on a linear model for microarray data [68]. *p*-values were calculated using an empirical moderated Bayes *t*-test with FDR (false discovery rate) correction for multiple testing. Criteria for selection of significantly up- or down-regulated miRNA were based on absolute fold change value greater than 2 and *p*-value < 0.05 with 20% of false discovery rate (FDR) correction.

mRNA expression data for target genes were extracted from our previous studies, where we examined mRNA profiles in adrenal glands from an identical research model. mRNA expression data for sham-operated male and female, gonadectomized and sex hormone replacement adrenals were downloaded from the GEO repository (N per group = 5, accession number: GSE93726). Identical cutoffs and data preparation for differential gene selection were used as in the case of miRNAs (abs (fold change) > 2 and *p* < 0.05 with 20% FDR correction, removing low variance genes). The obtained data were visualized using “ggplot2” and “ggprism” packages [69,70]. The overall profile of expression changes for both miRNA and mRNA was presented as volcano plots. Principal component analysis (PCA) of filtered miRNA and mRNA datasets was performed and visualized using the “factoextra” package [71].

### 4.5. miRNA-Target Gene Identification

To identify target genes for differentially expressed miRNAs, we used the “miRNAtap” package [72]. Sets of miRNAs assigned to individual clusters were used as a query for searching target genes in the following databases: for predicted targets—DIANA, Miranda, PicTar, TargetScan, and for experimentally validated targets—miRTar, miRWalk. For further analysis, we retrieved only targets that were identified from at least three databases. In the step of target filtration, we selected only target genes above the cutoff criteria for differentially expressed genes (abs (fold) > 2 *p* < 0.05 with 20% correction of FDR). For the final identification of miRNA-target genes, we left only those targets whose fold change was inversely correlated with the fold change in relevant miRNAs. This selected set of unique target genes was subjected to functional annotation and clusterization by the DAVID (Database for Annotation, Visualization, and Integrated Discovery) bioinformatics tool [73,74]. Gene symbols of differentially expressed genes were uploaded to DAVID by the “RDAVIDWebService” BioConductor package [75], where DEGs were assigned to relevant Gene Ontology (GO) terms, with subsequent selection of significantly enriched GO terms from the GO BP FAT database. The *p*-values of selected GO terms were corrected with the Benjamini–Hochberg correction, described as adjusted *p*-values [76]. Relevant GO ontological groups with adjusted *p*-values below 0.05 were visualized by bubble plot.

### 4.6. miRNA Co-Expression Analysis–Clustering of miRNA Data

A set of miRNA expression data whose expression was significantly regulated in at least one of the compared pairs was selected for analysis. To determine the optimal number of clusters, we applied a repeatedly calculated sum of squared error (SSE) measurement with an increasing number of clusters. K-means algorithm was used for clustering of miRNA expression profiles (according to the manual from: https://scienceparkstudygroup.github.io/rna-seq-lesson/08-cluster-analysis/index.html#6-clustering-using-k-means, accessed on 12 December 2024). Clustering was performed on the average expressions from each experimental group using “kmeans”-core R function

For each cluster, the centroid values and core miRNA sets were determined. Core miRNAs were generated by filtering the fitting level of miRNAs expression to centroid values where the miRNA expression profile displayed a high correlation to centroids for a given cluster (correlation > 0.8). Mean expression, normalized expression values, and fold changes for core miRNA of each cluster were visualized as a heatmap using the “Complexheatmap” package [77].

### 4.7. Comparative Analysis to Other Data

A comparative analysis of the miRNA expression profile obtained in the current study to the miRNA expression profile from similar experiments was carried out using two datasets deposited in Gene Expression Omnibus. First, miRNA deep sequencing data from 21 to 23 organs of five male and five female, 12–13 weeks old, Sprague Dawley rats. (GEO accession number: GSE78031, https://www.ncbi.nlm.nih.gov/geo/query/acc.cgi?acc=GSE78031, accessed on 12 December 2024). From the entire raw count sets, only those for adrenal glands were selected, and then differential expression analysis was performed using the “Deseq2” package [78]. As a cut-off criterion, *p* < 0.05 was used with 20% FDR correction.

The second analysis employed microarray miRNA data from rat adrenals of animals treated with vehicle (control), ACTH, 17α-ethinyl estradiol (17α-E2), or dexamethasone (DEX) (GEO accession number: GSE78031). CEL files for the control and 17α-ethinyl estradiol-treated groups were downloaded and subjected to downstream analysis. Expression analyses, including the identification of differential miRNAs, were performed in the same protocol as in our own experiments (except that the biological annotation of rat miRNAs was retrieved from the “pd.mirna 2.0” package).

From the obtained expression datasets, only miRNAs whose expression was significantly altered in at least one comparison in our analyses were filtered out. The result was presented on box plots (Figure 6A,B).

### 4.8. Validation of miRNA Expression by qPCR

QPCR was performed using the Lightcycler 2.0 instrument (ROCHE) with version 4.05 of the software. The miRCURY LNA RT Kit was used for reverse transcription, and the miRCURY LNA SYBR Green PCR Kit for QPCR, following the manufacturer’s protocols (Qiagen, Hilden, Germany). The expression of the studied miRNA was normalized to RNU1A1. The primer sets used were the miRCURY LNA miRNA PCR Assays (Qiagen, Hilden, Germany).

### 4.9. Statistical Analysis

The applied statistical analyses of miRNA profiling are part of the BioConductor packages. The RT-QPCR data are presented as the means of fold change, and the statistical significance of the differences between the compared groups was estimated using Student’s *t*-test.

## 5. Conclusions

The significance of sex hormones in normal adrenal function is unquestionable. Studies have shown that testosterone replacement significantly stimulates the expression of genes related to lipid and cholesterol metabolism, whereas estradiol inhibits the expression of corepressor genes in rat adrenal glands [7]. Additionally, in these animals, mRNA expression in adrenal cortical cells is sex-specific [6]. Furthermore, miRNA molecules regulate adrenal gene expression [38].

To the best of our knowledge, our study is the only one to date that has analyzed the effects of gonadectomy and sex hormone replacement on the regulation of miRNA expression in the rat adrenal gland. In both our previous and present studies, we observed the most significant effect on changes in miRNA and mRNA expression in the ORX + T vs. OVX + E comparison [7]. Moreover, we identified several new miRNAs and described changes in target gene expression and biological processes that are regulated by these miRNAs. This finding will aid in developing a more comprehensive understanding of adrenal physiology.

## Data Availability

All of the data discussed in this work, if not already included in the manuscript, are available from the corresponding author on reasonable request.
